# A null allele of granule bound starch synthase (*Wx-B1)* may be one of the major genes controlling chapatti softness

**DOI:** 10.1371/journal.pone.0246095

**Published:** 2021-01-28

**Authors:** Venkatesh Chunduri, Natasha Sharma, Monika Garg

**Affiliations:** Agri-Biotechnology Division, National Agri-Food Biotechnology Institute, Mohali, Punjab, India; Institute of Genetics and Developmental Biology Chinese Academy of Sciences, CHINA

## Abstract

Chapatti (unleavened flatbread) is a staple food in northern India and neighboring countries but the genetics behind its processing quality are poorly understood. To understand the genes determining chapatti quality, differentially expressed genes were selected from microarray data of contrasting chapatti cultivars. From the gene and trait association studies, a null allele of granule bound starch synthase (GBSS; *Wx-B1*) was found to be associated with low amylose content and good chapatti quality. For validation, near-isogenic lines (NILs) of this allele were created by marker assisted backcross (MAB) breeding. Background screening indicated 88.2 to 96.7% background recovery in 16 selected BC_3_F_5_ NILs. Processing quality and sensory evaluation of selected NILs indicated improvement in chapatti making quality. Traits that showed improvement were mouthfeel, tearing strength and softness indicating that the *Wx-B1* may be one of the major genes controlling chapatti softness.

## Introduction

Wheat is an important crop worldwide. In India, Nepal, Bangladesh, Pakistan, Sri Lanka, East Africa, and the Caribbean, it is mainly consumed in the form of an unleavened flatbread–the chapatti. Limited studies have been carried out to understand the genes/QTLs involved in chapatti making quality [1]. A good chapatti has white colour, less dough stickiness, easy to roll, soft pliable texture, soft chewing mouth feel and typical taste and aroma [2,3]. Major constituents of wheat determining end product quality are proteins and carbohydrates. Seed storage proteins in wheat are mainly glutenins and gliadins, which confer visco-elasticity and extensibility to the dough [4]. Glutenins are comprised of high molecular weight glutenin subunits (HMW-GS) and low molecular weight glutenin subunits (LMW-GS). HMW-GS constitute only 12% of total seed storage proteins but they determine 60% of the variation in baking properties [5]. LMW-GS constitute about 33% of total seed storage proteins and play an important role in bread-making quality by forming di-sulphide bridges with HMW-GS and helping in the formation of gluten polymer [6]. On the other hand, gliadins constitute 40–50% of total storage proteins but their effect on processing quality is limited [7,8]. Other than gluten proteins, puroindoline proteins (PINs) play an important role in grain texture and end-use quality [9]. A high amount of puroindoline proteins results in soft kernel texture. Milling of soft grains requires less force, thus there is less damaged starch and more intact starch granules, which decreases water absorption during dough making [10]. Hard textured wheat is more suitable for bread making while soft textured wheat is more suitable for the preparation of cookies as the intact starch granules impart crunchiness to the final product [11].

Other than proteins, starch (65–70% of the endosperm) also plays an important role in end-product quality. Different components of the starch, amylose (AM, 20–30%) and amylopectin (AP, 70–80%) and their ratios (AP/AM) influence properties like pasting, gelatinization and cooking quality [12–14]. Lower amylose content corresponds to greater water absorption capacity (and thus greater swelling power), higher peak viscosity of paste, lower peak viscosity temperature, lower final viscosity, greater resistance to retrogradation [12,15–17] and better noodle- and steamed bun-making quality [14,18,19].

Most reports on the end-product quality of wheat are related to bread, biscuit, and noodles. Chapatti, despite being the important food in several countries, has attracted limited attention [2,20–22]. Pre-green revolution cultivars like C306, C518, C591 and C273 had good chapatti quality but poor agronomic traits including tall stature, low grain yield and were prone to lodging. The introduction of semi dwarf, high yielding varieties during the green revolution led to the loss of chapatti quality traits in modern wheat varieties [23]. Our target was to create wheat lines with chapatti quality on par with pre-green revolution cultivars with grain yield at the level of post-green revolution cultivars. Previous research on chapatti quality focused on the comparison of chapatti making quality between cultivars [1,24], or the effect of additives on its quality [21,25]. Srivatsava [26] proposed that protein subunits have some influence but are not a major factor for determining chapatti quality. Other traits influencing chapatti quality have not been explored much.

In this study, we have screened the previously published microarray data generated from contrasting chapatti quality wheat cultivars [1]. From the gene and trait association studies, one gene with a positive influence on chapatti quality was selected. Crosses were made between good chapatti quality cultivar C306 and two high yielding Indian wheat varieties to create NILs using a backcross breeding method. Screening for yield and yield-related components as well as physicochemical, rheological and sensory parameters indicated that the selected gene is associated with chapatti softness. The current study highlights the usefulness of modern biotechnological tools like microarray for identification of gene of interest and its validation by the creation of NILs in a limited time.

## Materials and methods

### Screening of differentially expressed genes

For screening of differentially expressed genes and selection of candidate genes, seed microarray data of good (C306 and LOK1) vs. poor (WH291 and Sonalika) chapatti quality wheat cultivars (cv.), were collected at three developmental stages (7, 14, and 28 days after anthesis {DAA}), containing 61,290 probe sets representing about 25 K unigenes was used for screening [1].

### Screening of candidate genes

Based on microarray data granule bound starch synthase (GBSS), HMW-GS and puroindoline genes were selected as candidates for further analysis. Fourteen wheat cultivars with known chapatti quality (information provided by Punjab Agricultural University, Ludhiana, Punjab, India) were screened for allelic diversity. Polymorphism in the GBSS and puroindoline genes were screened by PCR based markers (Table 1). HMW-GS were screened using sodium dodecyl sulfate poly acrylamide gel electrophoresis [27].

**Table 1:** List of genes and primer sequences utilized under study.

S.No.	Gene	Location	Primer sequence	Reference
**1**	Wx-A1	7A	forward: 5’ CGTTTTAACTATACGTCTCGC 3’	[28]
			reverse: 5’ ATATGCAAAGGAGGTGAGGAAC 3’	
**2**	Wx-B1 (wild type)	4AS	forward: 5’ CTGGCCTGCTACCTCAAGAGCAACT 3’	[19]
			reverse: 5’ CTGACGTCCATGCCGTTGACGA 3’	
**3**	Wx-B1 (null)	4AS	forward: 5' CGTAGTAAGGTGCAAAAAAGTGCCACG 3'	[19]
			reverse: 5' ACAGCCTTATTGTACCAAGACCCATGTGTG 3'	
**3**	Wx-D1	7D	forward: 5’ CAGATCGAATGCCGGTACC 3’	[28]
			reverse: 5’ CGCAAAATTGATATGCCTGTT 3’	
**4**	WMC313	4AS	forward: 5’ GCGGTCGTCTATTAATCTGACG 3’	[29]
			reverse: 5’ GGGTCCTTGTCTACTCATGTCT 3’	
**5**	*Pina-D1*	5DS	forward: 5′ CATCTATTCATCTCCACCTGC 3’	[30]
			Reverse: 5’ GTGACAGTTTATTAGCTAGT 3′	
**6**	*Pinb-D1*	5DS	forward: 5′ AATAAAGGGGAGCCTCAACC 3′	[30]
			Reverse: 5’ GAATAGAGGCTATATCATCACCA 3′	

Seeds of all the fourteen cultivars were also screened for amylose content. The wheat starch granules were isolated according to reference [31]. The percentage amylose content in the starch granule pellet was determined by using the Megazyme amylose/amylopectin assay kit [32].

### Near isogenic lines (NILs) development

**Fig 1:**
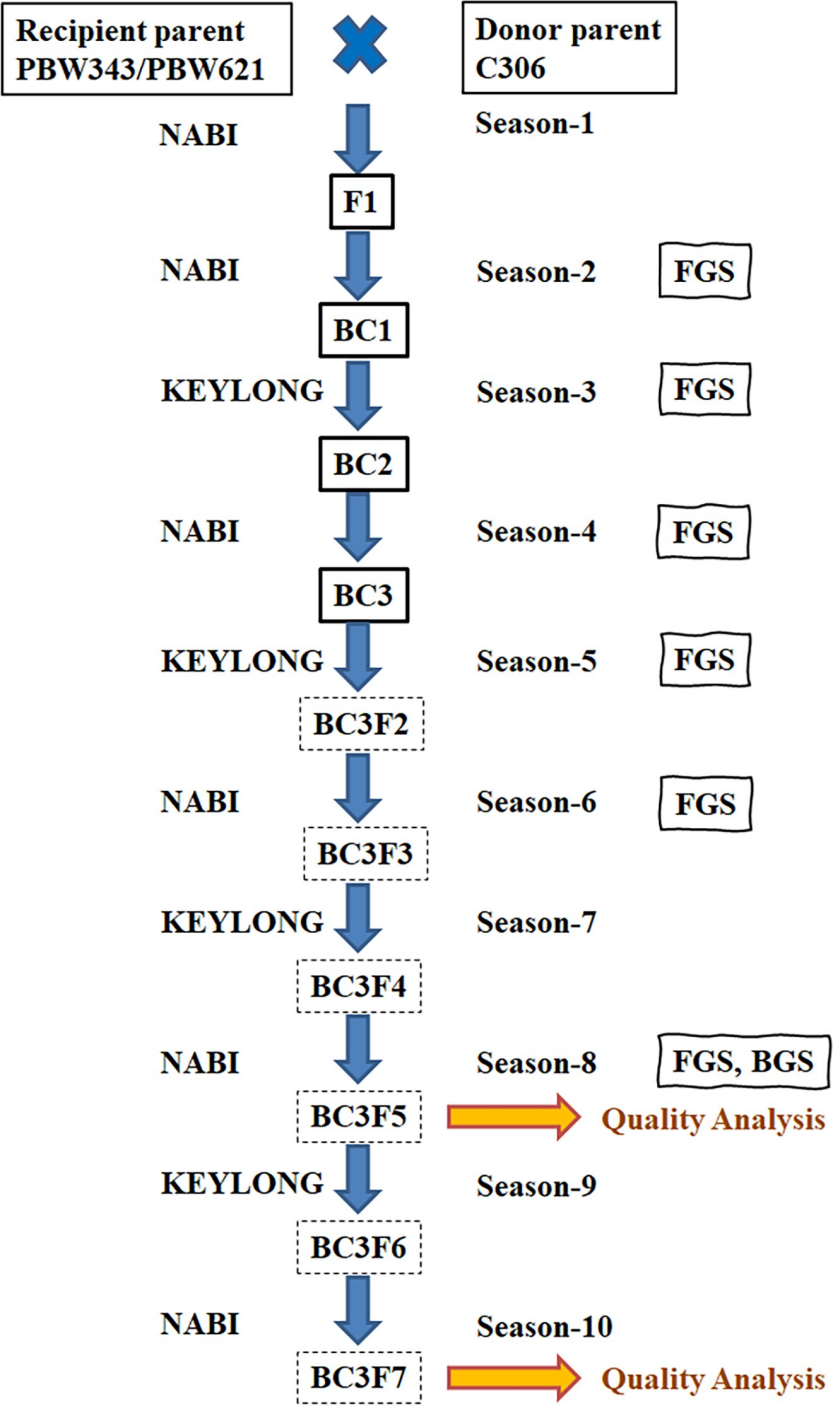
Schematic representation of the crossing program for the generation of NILs for null allele of GBSS-4A gene. Solid box indicates crossing and the dotted box indicates selfing. FGS- Foreground selection; BGS- Background screening.

The NILs were developed by crossing the good chapatti quality wheat cv C306 as the donor, with poor chapatti quality but high yielding cultivars PBW343and PBW621 as recipients, through the backcross breeding method (Fig 1). All wheat cultivars/lines were grown in the farms of National Agri-Food Biotechnology Institute, Mohali, Punjab, India (30°44’10” N Latitude at an elevation of 351 m above sea level) during the main season and at Indian Institute of Wheat and Barley Research at Dalang Maidan, Himachal Pradesh, India (32˚30’27.9” N Latitude and 76˚59’34” E Longitude at an elevation of 2971 m above sea level) in the offseason. Three backcrosses (BC_3_), were followed by seven selfing generations. The BC_3_ were named as C3 (from the cross C306/4*PBW343) and C6 (from the cross C306/4*PBW621). Individual selections (plants/plots) from the same cross were number from A to Z e.g. C3A to C3H from cross C3 and C6A to C6H from cross C6. The foreground selection (FGS) was carried out using SSR marker WMC313 followed by co-dominant marker *Wx-B1*. The background screening (BGS) was carried out using 400 deletion bin-based primers, spread across 42 chromosomes [33].

### Grain quality parameters

Sodium dodecyl sulfate sedimentation (SDSS) test was performed on a small scale using 1 g flour [34]. Dough extensibility tests were performed on texture analyzer (Stable Microsystems) using Kieffer dough and gluten extensibility rig. Peak positive force, stretching distance, and area to positive peak were measured [35]. Solvent retention capacity (SRC) tests were performed according to American Association of cereal chemists (AACC) method 56–11.02 for deionized water, sucrose solution (50% w/w), sodium carbonate solution (5% w/w), and lactic acid solution (5% w/w). Thermal properties of starch (isolation as mentioned above) were estimated using differential scanning calorimeter (DSC; 822, Mettler Toledo, Columbus, OS, USA) equipped with a thermal analysis data station. Onset temperature (To), peak temperature (Tp), conclusion temperature (Tc) and enthalpy (ΔH) were calculated using STARe software for thermal analysis (STARe SW 9.01).

### Chapatti sensory evaluation and texture analysis

Sensory evaluation of fresh chapattis was done by a panel of 10 members. The sensory attributes of chapatti were evaluated in terms of dough stickiness, rollability, puffing height, black spots, color, taste, aroma, mouth feel, tearing strength and softness by a panel consisting of ten members using the 9 point-Hedonic scale with a score of 9—extreme liking, 8—like very much, 7—like moderately, 6—like slightly, 5—neither like nor dislike, 4—dislike slightly, 3—dislike moderately, 2—dislike very much and 1 for extreme disliking [36]. Donor cultivar C306 was used as the positive control. Chapatti tensile strength was estimated on the texture analyzer (Stable Micro systems) within 1hr of baking, and tensile modulus (TM) was measured [37] as:

TM(MPa)=Tensilestress/Tensilestrain=(F/A)/(ΔL/L)

Where, F—peak force to rupture, A—cross-section area (m^2^), L—initial chapatti length (m) and ΔL—change in length (extensibility).

### Statistical analysis

Results were analyzed using one-way analysis of variance (ANOVA) followed by Tukey’s-b test using IBM SPSS Statistics 21.0. The Principal Component Analysis was performed by XLSTAT 2020 for the analysis of chapatti quality, PIN genes, HMW-GS, null *Wx-B1*, starch content.

## Results

### Short listing of candidate genes

For candidate gene screening differential expression microarray data of good vs. poor chapatti quality lines was utilized [1]. This data indicated differential expression of genes like gliadins and glutenins, GBSS-I, peroxidase, proteinase, amylases, puroindolines, etc. [1]. Three genes and their isoforms namely GBSS-I, HMW-G and puroindoline were shortlisted based on their differential expression and previous processing quality related literature support (S1 Table).

### Selection of candidate genes

**Fig 2:**
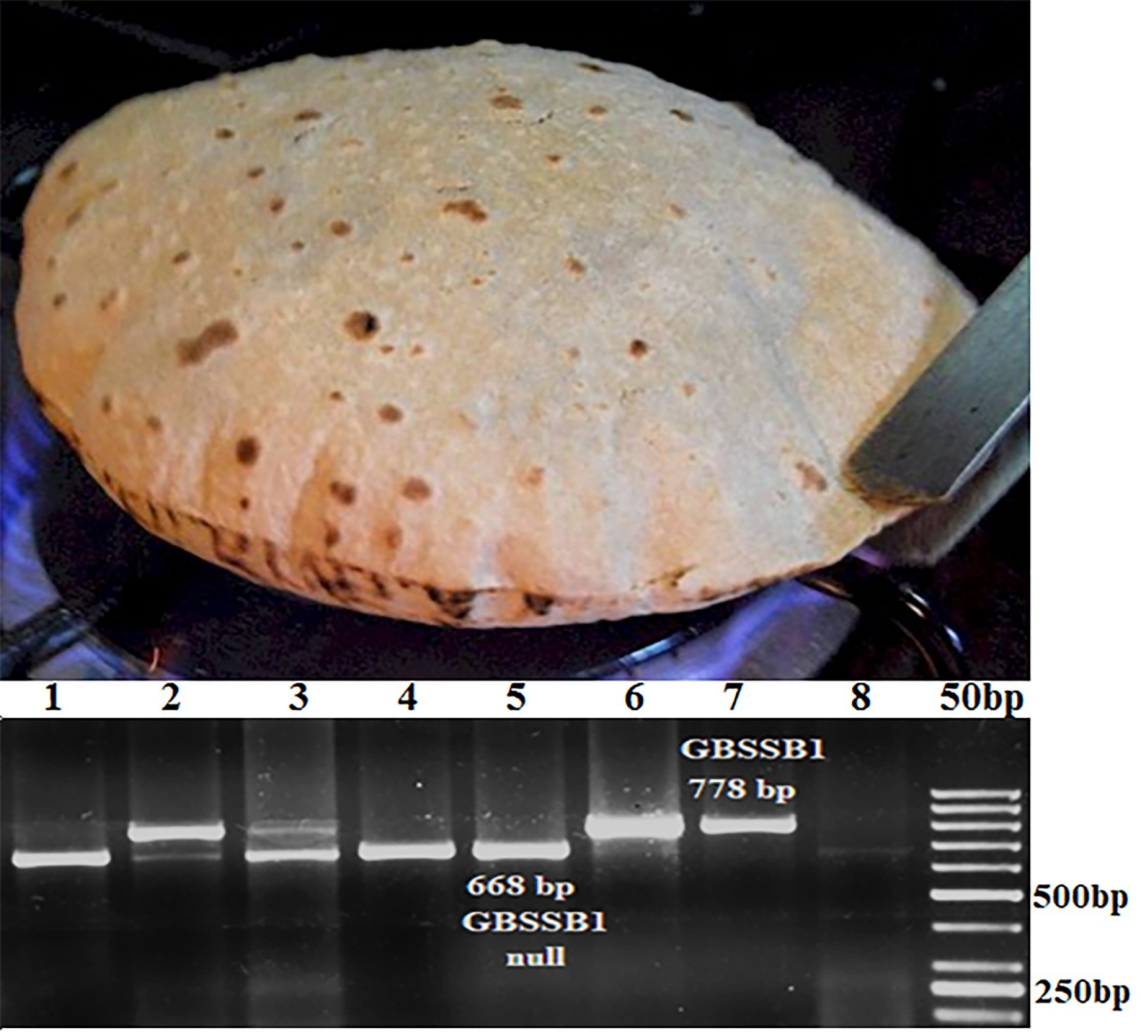
A. Good chapatti with small uniform spots and good puffiness. B Screening of Indian germplasm with codominant PCR markers to understand the variation of GBSS-4A allele. The 668 bp fragment indicates null allele while 778 bp fragment indicates functional allele. 1. C306; 2. Sonalika; 3. K8027; 4. LOK1; 5. HI1563; 6. PBW343; 7. PBW621; 8. Negative control.

The good chapatti lines (Table 2) had hard seed texture with the hardness index 74–95. Poor chapatti lines were also hard with hardness index 70–82. While very poor (S. No. 13–14; Table 2) lines had soft grain with hardness index 30–40. In the case of puroindoline genes (*Pina* and *Pinb*), all the tested hard wheat lines (1–12; Table 2) had non-functional *Pina* allele *Pina-D1b* and functional *Pinb* allele *Pinb-D1a* with exception of one, cv. Sonalika (S. No. 11; Table 2) with functional *Pina-D1a* and non-functional *Pinb-D1b* alleles. Soft wheat lines (S. No. 13–14; Table 2) had both functional *Pina-D1a* and *Pinb-D1a* alleles. In the case of HMW-GS genes observed allelic variation was *null*, *1*, 2* at the locus *Glu-1A*; *7*, *7+8*, *7+9*, *17+18*, *20* at locus *Glu-1B*; *5+10*, *2+12* at locus *Glu-1D* (Table 2). In the case of GBSS-I genes, *Wx-A1* and *Wx-D1* were non-polymorphic (S1 Fig). The *Wx-B1* had non-functional null allele (A; Table 2; 668 bp; Fig 2) in good chapatti lines and functional allele (P; Table 2, 778 bp; Fig 2) in the poor chapatti lines. The screening revealed amylose starch content between 29.1 and 30.3 in poor chapatti lines and 25.6 and 26.8 in good chapatti lines. One exception was Lok1 with good chapatti quality and amylose content of 29.3. The Pearson correlation matrix also showed that the chapatti quality is positively correlated with grain hardness (0.837) and null *Wx-B1* (0.9) and negatively correlated with amylose content (-0.765) and *Pina-D1a* (-0.785) (S2 Table). As *Pina-D1a* determines grain hardness and contribution of grain hardness towards chapatti quality was already known, null *Wx-B1* was selected for further validation by preparing NILs.

**Table 2:** Screening of wheat cultivars with contrasting chapatti making quality for understanding the variation in selected traits.

S. No.	Cultivar	Chapatti quality	Grain texture	Grain hardness index	*PINa -D1*	*PINb -D1*	HMW-GS *Glu-1A*	HMW-GS *Glu-1B*	HMW-GS *Glu-1D*	*Wx-B1*	Amylose content
**1**	PBW550	Good	Hard	78.5	b	a	2*	7	5+10	A	26.0
**2**	C306	Good	Hard	95	b	a	Null	20	2+12	A	25.6
**3**	LOK1	Good	Hard	74.2	b	a	2*	17+18	2+12	A	29.3
**4**	PBW175	Good	Hard	83	b	a	2*	7+8	2+12	A	26.8
**5**	HI1563	Good	Hard	75	b	a	2*	7+8	2+12	A	26.4
**6**	K8027	Good	Hard	91	b	a	2*	17+18	5+10	A	26.2
**7**	HD2888	Good	Hard	85	b	a	Null	20	2+12	A	25.9
**8**	HI1500	Good	Hard	90	b	a	Null	20	2+12	A	26.3
**9**	PBW343	Poor	Hard	80	b	a	1	7	5+10	P	29.8
**10**	PBW621	Poor	Hard	77.3	b	a	2*	17+18	2+12	P	30.3
**11**	Sonalika	Poor	Hard	70	a	b	2*	7+9	2+12	P	29.1
**12**	WH291	Poor	Hard	82.3	b	a	2*	20	2+12	P	29.9
**13**	Chinese Spring	Very Poor	Soft	38.6	a	a	Null	7+8	2+12	P	29.2
**14**	IITR67	Very Poor	Soft	39	a	a	Null	7+8	2+12	P	29.7

### Development of NILs

Around 70 BC_1_ plants from each cross were screened by co-dominant gene specific marker for heterozygosity of the *Wx-B1* gene (foreground selection) and a linked SSR marker. Selected plants were marked and used for backcrossing. Around 70 plants from BC_2_ and BC_3_ were screened. Total of 150 plants were screened at BC_3_F_2_ stage. 20 selected plants each from crosses C3 and C6 were advanced to BC_3_F_4_ stage (Table 3). FGS these 40 BC_3_F_4_ lines were showing null Wx-B1 allele. They were also checked for uniformity and morphological similarity with the recipient parent. A total of 16 BC_3_F_5_ plots in the subsequent year were selected (8 from PBW343 cross {C3A to C3H} and 8 from PBW621 cross {C6A to C6H} for grain quality parameters, chapatti sensory evaluation, generation advancement and background screening (Year 1 data). As it was BC_3_ based progeny, around 93.75% background recovery was expected. Among the 16 lines analyzed, background recovery was found to be between 88.2 and 96.7%. Based on the above-mentioned parameters, 2 BC_3_F_7_ NILs, one from each cross with maximum recipient parent recovery (96.7% and 96.3%) were chosen for next year field performance and quality study (Year 2 data). The yield (ton/hectare) of NILs *viz*., C3C (2.68) and C6H (4.77) were significantly higher than donor C306 (1.3) and lower than the recipient parents PBW343 (3.3) and PBW621 (5.8) (S3 Table). The thousand-kernel weight was similar in NILs, donor and recipient cultivars. HMW-GS and puroindoline alleles of NILC3C and NILC3H were similar to their recipient parents (S3 Table).

**Table 3:** Screening and selection of backcross generations for preparation of NILs.

Generation	C306/4*PBW343			C306/4*PBW621	
	Screening method	No. of plants/lines screened	Positive plants/lines selected after FGS/BGS	No. of plants/lines screened	Positive plants/lines selected
**BC**_**3**_**F**_**3**_	FGS	75	20	75	20
**BC**_**3**_**F**_**5**_ **(Year 1)**	FGS, BGS, Quality traits	20	8/1	20	8/1
**BC**_**3**_**F**_**7**_ **(Year 2)**	Quality traits	1	1	1	1

FGS = Foreground selection, BGS = Background screening.

### Evaluation of grain quality parameters of NILs

#### Sodium dodecyl sulphate sedimentation value (SDSS)

In year 1, SDSS of 8 selected NILs from each cross ranged between 2.43 to 3.5 for C3 NILs and 3.63 to 7.07 for C6 NILs. For donor C306 it was 2.27 and for recipients PBW343 and PBW621, it was 6.6 to 9.7, respectively (S4 Table). In year 2, SDSS of selected NILs, donors and recipients were in the range of 2.8–6.13 (S2 Fig). NILs had significantly lower SDSS value than their recipient parents. In year 2, NILC3C had significantly lower SDSS value than the donor C306 (S2 Fig).

#### Dough extensibility test

Dough extensibility test in the year 1, revealed that the separation distance, peak positive force and area to positive peak were significantly higher in recipient cultivars as compared to the donor with the highest values observed in PBW621. The NILs showed transgressive segregation i.e., some of NILs had values even lower than C306 and others higher than PBW621 (S5 Table). In year 2, a similar trend was observed for parents. For NILs these values were lower than their recipient parents (S3 Fig). In year 2, these values in NILC3C were even lower than C306 (S3 Fig).

#### Solvent retention capacity (SRC)

Recipient parents had significantly higher SRC than C306 in all the four types of solutions, but SRC of NILs followed a random pattern, neither aligning with donor or recipients (S6 Table).

#### Differential scanning calorimetry (DSC)

We observed that the To, Tp, Tc of donor line C306 and NILC3C were significantly higher than PBW343. But there was no significant difference observed in ΔH of all the three lines. The To and Tc values of PBW621 were lower than C306 and NILC6H. There was no significant difference observed in ΔH of all the three lines. (Table 4). Overall, there was an increase in either or all of To/Tp/Tc in the donor parent C306 and NILs.

**Table 4:** Thermal properties determined by DSC for NILs and parents (year 2).

Sample ID	To	Tp	Tc	ΔH
**NILC3C**	58.56±0.01^b^	62.74±0^b^	67.36±0^b^	6.83±0.66^a^
**C306**	58.54±0.05^b^	62.03±0.13^b^	66.84±0.06^b^	6.57±1.04^a^
**PBW343**	57.07±0.37^a^	60.96±0.17^a^	65.52±0.19^a^	5.07±1.92^a^
**NILC6H**	58.33±0.08^ab^	61.98±0.07^a^	66.09±0.14^b^	7.42±1.05^a^
**C306**	58.54±0.05^b^	62.03±0.13^a^	66.84±0.06^c^	6.57±1.04^a^
**PBW621**	57.43±0.24^a^	61.22±0.18^a^	65.33±0.11^a^	6.54±1.31^a^

Data was represented in mean ± SE of 6 replicates.

Same letters depict they are not significantly different (p<0.05).

### Evaluation of chapatti quality of NILs

In year-1, most of the NILs showed significantly better chapatti sensory evaluation than their recipient parents (C306>NILs>Recipients) (S7 Table). NILC3C, NILC6H showed better chapatti sensory evaluation when compared to other NILs. In the year-2, the selected NILs showed statistically better chapatti compared to their recipient parents but similar to/lower than C306 (Table 5). The parameters that were consistent for both NILs and showed major difference were chapatti mouthfeel, tearing strength and softness. These traits were similar to the donor C306. Some parameters like rollability and puffing were better for NILC3C and black spot, color and taste were better for NILC6H.

**Table 5:** Chapatti sensory parameters of selected NILs in comparison to parents (year 2).

Sample ID	Stickiness	Roll-ability	Puffing	Black spots	Color	Taste	Aroma	Mouth-feel	Tearing	Softness	Total Score	TM at 0’ (M Pa)
**NILC3C**	8.6±0.16^b^	8.6±0.16^b^	8.5±0.22^b^	7.9±0.28^b^	7.7±0.3^b^	7.9±0.23^b^	8.3±0.15^b^	8.7±0.15^b^	8.8±0.13^b^	8.7±0.15^b^	83.7±0.92^b^	0.19±0.01^a^
**C306**	8.8±0.13^b^	8.8±0.13^b^	8±0.26^b^	8.5±0.27^b^	8.4±0.16^b^	8.8±0.13^c^	8.9±0.1^c^	8.8±0.13^b^	8.9±0.1^b^	8.8±0.13^b^	86.7±1.14^c^	0.21±0^a^
**PBW343**	6±0.21^a^	6.2±0.29^a^	6±0.21^a^	6±0.15^a^	5.8±0.25^a^	6±0.21^a^	7.1±0.18^a^	6.6±0.16^a^	5.8±0.13^a^	7±0.26^a^	62.5±0.58^a^	0.28±0.02^b^
**NILC6H**	8.6±0.16^b^	8.2±0.13^b^	7.7±0.15^b^	8.4±0.16^b^	8.2±0.13^b^	8.3±0.15^b^	7.7±0.15^b^	8.4±0.16^b^	8.7±0.15^b^	8.7±0.15^b^	83.9±0.78^b^	0.31±0.04^b^
**C306**	8.8±0.13^b^	8.8±0.13^b^	8±0.26^b^	8.5±0.27^b^	8.4±0.16^b^	8.8±0.13^c^	8.9±0.1^b^	8.8±0.13^b^	8.9±0.1^b^	8.8±0.13^b^	86.7±1.14^c^	0.21±0^a^
**PBW621**	7.8±0.2^a^	7±0.3^a^	6.7±0.15^a^	6.3±0.15^a^	6.7±0.26^a^	5.8±0.2^a^	6.3±0.15^a^	6.5±0.22^a^	7.2±0.2^a^	6.9±0.28^a^	67.2±0.53^a^	0.38±0.02^b^

TM- Tensile Modulus. Data was represented in mean ± SE of 10 replicates. Same letters depict they are not significantly different (p<0.05).

TM was significantly lower in C306 compared to recipient parents (Table 4). The TM of NILC3C is similar to that of C306 and NILC6H was intermediate between C306 and recipient.

## Discussion

This study utilized microarray data to shortlist genes related to chapatti making quality based on differential expression data of contrasting lines. Out of several differentially expressed genes, a few genes with more than 10-fold differential expression along with previous literature support were shortlisted for further validation. Several studies on differential expression of contrasting trait lines have been reported, and these studies have identified several differentially expressed genes belonging to diverse pathways and highlighted complex mechanisms involving regulation at epigenomic, transcriptional, translational and post-translational levels [38–42], but shortlisting of a few candidate genes and their validation has not been reported. Although transcriptional level control is the major one, sometimes changes at this level may not be reflected at protein accumulation level [42–44], but still, transcriptional level control is a valuable resource for careful utilization.

In this study, we shortlisted three genes (GBSS, puroindolines, HMW-GS) and their isoforms, screened them on previously known chapatti quality lines, selected GBSS-4A, prepared its NILs and determined its influence on chapatti softness. These three are major genes reported for their association with wheat processing into different food products like bread, biscuits, noodles etc. One of these three, the puroindoline genes are responsible for grain texture. The soft wheat with low protein content is used for biscuits and cakes and hard wheat with high protein content is preferred for bread and noodles. We could find an association of grain hardness with chapatti quality, as soft wheat chapattis with both functional puroindoline alleles (*Pina-D1a* and *Pinb-D1a*) made very poor chapattis. Effect of grain hardness on chapatti quality has not been reported much in scientific publications [45], as the major area of Indian sub-continent consuming chapattis, grows hard wheat. But, its effect on chapatti quality was long understood at the time of the green revolution, when dwarf, soft, red and high yielding wheat varieties were introduced in India and Pakistan by the CIMMYT, Mexico. Those varieties had significantly higher yield but were rejected by the population as these made hard chapattis and correlated it with red color. This led to the selection of hard white CIMMYT lines and their wide adoption by the farmers and integration of these criteria in the wheat variety selection program of the country. The hard grain texture is pre-requisite for further investigation of candidate genes associated with chapatti quality. Thus, the recipients selected in this study were hard with high grain hardness index. The next gene studied was HMW-GS. It produced 4–5 subunits belonging to *Glu-A1* (0–1 subunits) on chromosome 1A, *Glu-B1* (1–2) on chromosome 1B and *Glu-D1* (2) on chromosome 1D and previous studies have shown co-relation of their allelic variation with bread-making quality [5]. We could not find their co-relation with chapatti making quality and same alleles were present in good as well as poor chapatti lines. Previously, alleles *Glu-D1xy-*5+10 [26] and *Glu-B1x-20* [46] have been reported to be associated with good chapatti quality in comparison to poor chapatti for *Glu-D1xy-*2+12 and other *Glu-B1xy* alleles. But conflicting results indicating no association of individual HMW-GS with chapatti making quality have also been reported [47,48]. This might be due to limited number of varieties used to study the correlation between the complex composition of HMW-GS and chapatti quality.

Our next gene of interest was GBSS-I, that includes three genes *Wx-A1*, *Wx-B1* and *Wx-D1* on chromosome 7A, 4A and 7D. Our polymorphism check of Indian germplasm indicated polymorphism at *Wx-B1* locus and its allelic variation was not only associated with variation in amylose content but also chapatti making quality, that was further confirmed by preparing and analyzing NILs of null allele of *Wx-B1* locus. Observations on rheological parameters like SDSS test, dough extensibility, SRC and DSC indicated that these parameters had an improvement in NILs compared to recipients. The SDSS value and dough extensibility of NILC3C were even lower than donor C306. It is envisaged that these might influence good chapatti rollability that was also found to better in this line. Contrasting reports of the positive and negative influence of SDSS on chapatti quality have been reported [22,49,50]. Higher dough extensibility has been related to easy sheeting/rolling of chapatti dough [22,51] and good chapatti quality. Thus, selected NILs had better ease of extending the dough. Higher values of Na_2_CO_3_ SRC in NILs indicated higher water absorption capacity of NILs as compared to the recipient, that is associated with good chapatti quality [52]. Observations on DSC results indicated an overall increase in either or all of To/Tp/Tc in the NILs in comparison to recipient parent and that might be due to lower amylose content. Chapatti quality evaluation indicated that selected NILs had statistically better chapatti quality than recipient parents but their score could not reach up to the level of C306. The positive influence was observed in the case certain parameters like the mouthfeel, tearing strength and softness. These traits were similar to the donor C306, indicating the association of these parameters to chapattis softness. This observation was supported by lower TM of NILs that is associated with softer chapattis with high extensibilities [20].

Preparation of NILs by marker assisted breeding is a long process that requires several generations, but with the estimation of background recovery, this period can be reduced. We used BC_3_ lines with background recovery of 96.7% and 96.3% as compared to the expected value of 93.75%. The NILs also have an advantage over RILs that are used for studying the association of trait of interest with the genes/genic region, as with careful selection these can be directly used for cultivation. NILs are most useful as it allows measurement for the effect of allelic variation at single candidate genes while eliminating background genetic variation [53].

Better mouthfeel, tearing strength, softness and dough extensibility observed in selected NILs indicate improved chapatti making quality. A similar observation has been documented for softness associated with Japanese Udon noodles that are also associated with a null allele of the same gene [15,54]. Udon with a firm surface and soft inside is preferred, similar are the requirement for chapatti making, firm surface for inhibiting it from sticking to the hot plate (tawa) and thus giving uniform black spots and soft inside to give it a soft mouthfeel. Therefore, GBSS-4A can be one of the major genes determining chapatti softness in hard wheat.

## Conclusions

The present study was aimed to identify traits associated with chapatti quality. The microarray expression analysis helped in shortlisting candidate genes and further studies helped in the selection of single genes. The NILs of this gene generated by marker assisted backcross breeding showed improvement in traits like mouthfeel, tearing strength and softness indicating GBSS-4A may be one of the major genes controlling chapatti softness.

## Supporting information

S1 Fig Gel-image of GBSS isoforms between Indian cultivars. 1. PBW550, 2. C306, 3. PBW343, 4. K8027, 5. PBW621, 6. Lok1, 7. WH291, 8. Sonalika, 9. 50 bp Marker.
(DOCX)

S2 Fig Graph showing the SDS sedimentation values of NILs in comparison with parents. (DOCX)

S3 Fig Graph showing the dough extensibility parameters of NILs in comparison with parents. (DOCX)

S1 Table Differential expression of candidate probes identified for processing quality at three different developmental stages. (DOCX)

S2 Table Pearson correlation matrix between chapatti quality and candidate traits. (DOCX)

S3 Table Patterns of puroindolines and HMW-GS, Yield relate parameters of selected NILs in comparison with parents (year 2). (DOCX)

S4 Table SDS sedimentation of NILs in comparison with parents (year 1). (DOCX)

S5 Table Dough extensibility of NILs in comparison with parents (year 1). (DOCX)

S6 Table SRC of NILs in comparison with parents (year 1). (DOCX)

S7 Table Chapatti sensory parameters of NILs in comparison with parents (year1). (DOCX)

S1 File (DOCX)
