# Supplementary material for: A null allele of granule bound starch synthase (Wx-B1) may be one of the major genes controlling chapatti softness
Source: PLoS One. 2021 Jan 28;16(1):e0246095. doi: 10.1371/journal.pone.0246095 (PMC7842929; doi:10.1371/journal.pone.0246095)
Supplement: S1 Fig — 1. PBW550, 2. C306, 3. PBW343, 4. K8027, 5. PBW621, 6. Lok1, 7. WH291, 8. Sonalika, 9. 50 bp Marker. (DOCX) [file pone.0246095.s001.docx]

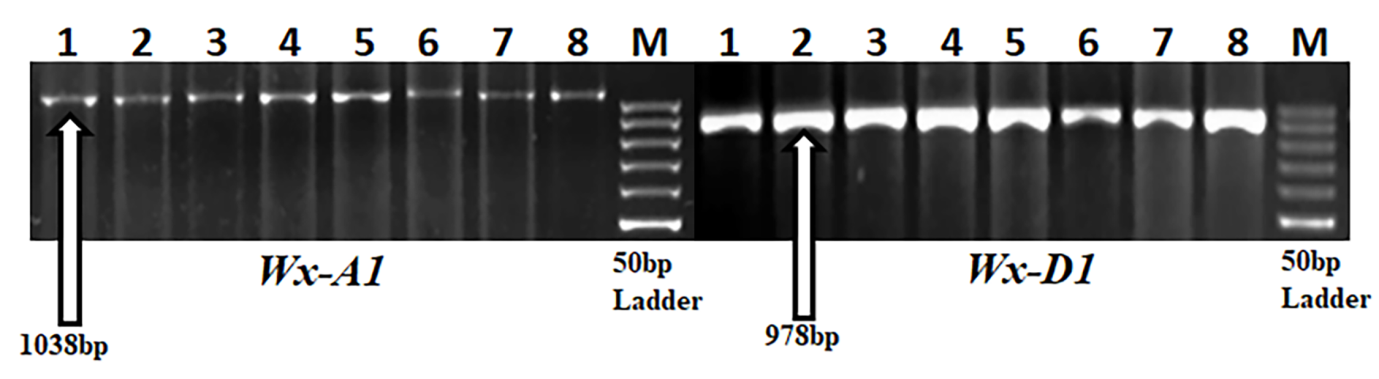


**S1 Fig. Gel-image of GBSS isoforms between Indian cultivars.** 1. PBW550, 2. C306, 3. PBW343, 4. K8027, 5. PBW621, 6. Lok1, 7. WH291, 8. Sonalika, 9. 50 bp Marker
