# Supplementary figures and images for: A null allele of granule bound starch synthase (Wx-B1) may be one of the major genes controlling chapatti softness
Source: PLoS One. 2021 Jan 28;16(1):e0246095. doi: 10.1371/journal.pone.0246095 (PMC7842929; doi:10.1371/journal.pone.0246095)

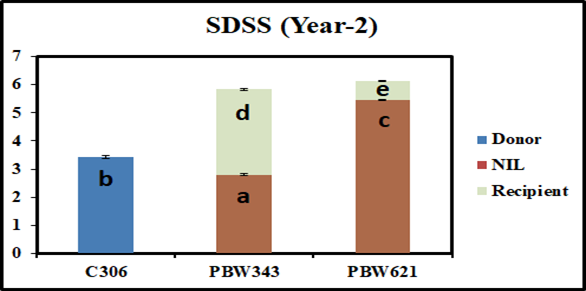


**S2 Fig. Graph showing the SDSS values of NILs in comparison with parents.**

Supplement: S2 Fig — (DOCX) [file pone.0246095.s002.docx]

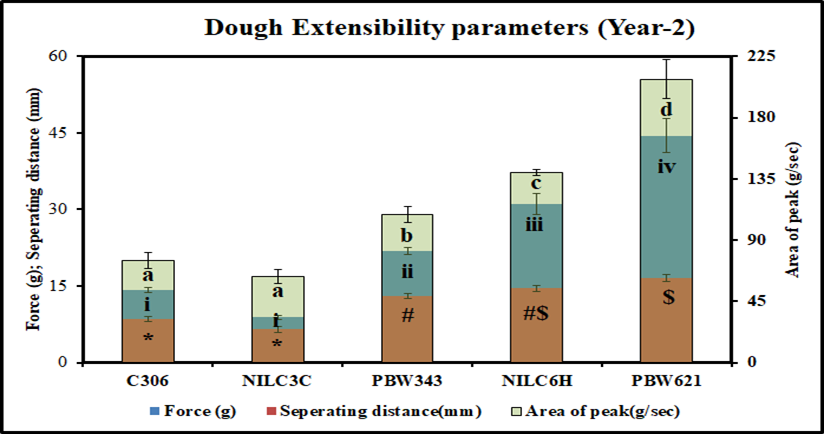


**S3 Fig. Graph showing the dough extensibility parameters of NILs in comparison with parents.**

Supplement: S3 Fig — (DOCX) [file pone.0246095.s003.docx]
