# Supplementary material for: A null allele of granule bound starch synthase (Wx-B1) may be one of the major genes controlling chapatti softness
Source: PLoS One. 2021 Jan 28;16(1):e0246095. doi: 10.1371/journal.pone.0246095 (PMC7842929; doi:10.1371/journal.pone.0246095)
Supplement: S1 Table — (DOCX) [file pone.0246095.s004.docx]

**S1Table .** Differential expression of candidate probes identified for processing quality at three different developmental stages.

| **Probe set ID** | **Putative gene function** | **7 DAA** | | **14 DAA** | | **28 DAA** | |
| --- | --- | --- | --- | --- | --- | --- | --- |
|  |  | **Fold change** | **Regulation** | **Fold change** | **Regulation** | **Fold change** | **Regulation** |
| **Ta.24114.7.A1_at** | Granule-bound starch synthase I | 6.7 | down | 26.9 | down | 92.8 | down |
| **Ta.24298.1.S1_x_at** | HMW-glutenin subunit Dx5 | 56.7 | up | 1.1 | down | 1 | up |
| **Ta.23141.1.S1_at** | Puroindoline a protein | 1.3 | down | 1.8 | down | 22.2 | up |
| **Ta.115.1.S1_at** | Puroindoline b protein | 34.8 | up | 1.2 | down | 1.1 | up |
