# Supplementary material for: A null allele of granule bound starch synthase (Wx-B1) may be one of the major genes controlling chapatti softness
Source: PLoS One. 2021 Jan 28;16(1):e0246095. doi: 10.1371/journal.pone.0246095 (PMC7842929; doi:10.1371/journal.pone.0246095)
Supplement: S2 Table — (DOCX) [file pone.0246095.s005.docx]

**S2 Table.** Pearson Correlation Matrixbetween chapatti quality and candidate traits.

| **Variables** | **Chapatti**  **quality** | **Grain hardness index** | **PINa-D1** | **PINb-D1** | **HMW-GS Glu-1A** | **HMW-GS Glu-1B** | **HMW-GS Glu-1D** | **null Wx-B1** | **Amylose content** |
| --- | --- | --- | --- | --- | --- | --- | --- | --- | --- |
| **Chapatti quality** | **1.000** | **0.837** | **-0.785** | 0.163 | -0.313 | 0.314 | 0.171 | **0.906** | **-0.765** |
| **Grain hardness index** | **0.837** | **1** | **-0.840** | 0.095 | -0.152 | 0.495 | 0.239 | **0.585** | **-0.548** |
| ***Pina-D1*** | **-0.785** | **-0.840** | **1** | -0.531 | 0.139 | -0.312 | -0.273 | **-0.603** | 0.427 |
| ***Pinb-D1*** | 0.163 | 0.095 | -0.531 | **1** | 0.221 | 0.040 | 0.145 | 0.320 | -0.190 |
| **HMW-GS Glu-1A** | -0.313 | -0.152 | 0.139 | 0.221 | **1** | -0.116 | 0.139 | -0.230 | 0.068 |
| **HMW-GS Glu-1B** | 0.314 | 0.495 | -0.312 | 0.040 | -0.116 | **1** | -0.431 | 0.224 | -0.170 |
| **HMW-GS Glu-1D** | 0.171 | 0.239 | -0.273 | 0.145 | 0.139 | -0.431 | **1** | 0.101 | -0.166 |
| **null *Wx-B1*** | **0.906** | **0.585** | **-0.603** | 0.320 | -0.230 | 0.224 | 0.101 | **1** | **-0.871** |
| **Amylose content** | **-0.765** | **-0.548** | 0.427 | -0.190 | 0.068 | -0.170 | -0.166 | **-0.871** | **1** |

Values in bold are different from 0 with a significance level alpha=0.05. Values in bold within the same factor indicate the variable with the largest correlation
