# Supplementary material for: A null allele of granule bound starch synthase (Wx-B1) may be one of the major genes controlling chapatti softness
Source: PLoS One. 2021 Jan 28;16(1):e0246095. doi: 10.1371/journal.pone.0246095 (PMC7842929; doi:10.1371/journal.pone.0246095)
Supplement: S3 Table — (DOCX) [file pone.0246095.s006.docx]

**S3 Table.** Patterns of puroindolines and HMW-GS, Yield relate parameters of selected NILs in comparison with parents (year 2).

| **Year 2** | | | | | | | |
| --- | --- | --- | --- | --- | --- | --- | --- |
| **Sample ID** | ***PINa -D1*** | ***PINb -D1*** | **HMW-GS *Glu-1A*** | **HMW-GS *Glu-1B*** | **HMW-GS *Glu-1D*** | **Yield (tonne/hectare)** | **TKW** |
| **NILC3C** | b | a | Null | 7 | 5+10 | 2.68±0.06^b^ | 41.81±0.02^a^ |
| **C306** | b | a | Null | 20 | 2+12 | 1.3±0.07^a^ | 42.33±0.51^a^ |
| **PBW343** | b | a | 1 | 7 | 5+10 | 3.3±0.22^b^ | 40.7±0.03^a^ |
| **NILC6H** | b | a | 2* | 17+18 | 2+12 | 4.77±0.23^b^ | 42.98±0.01^a^ |
| **C306** | b | a | Null | 20 | 2+12 | 1.3±0.07^a^ | 42.33±0.51^a^ |
| **PBW621** | b | a | 2* | 17+18 | 2+12 | 5.8±0.17^c^ | 41.68±0.44^a^ |

Data was represented in mean ± SE of 3 replicates. Same letters depict they are not significantly different (p<0.05)
