# Supplementary material for: A null allele of granule bound starch synthase (Wx-B1) may be one of the major genes controlling chapatti softness
Source: PLoS One. 2021 Jan 28;16(1):e0246095. doi: 10.1371/journal.pone.0246095 (PMC7842929; doi:10.1371/journal.pone.0246095)
Supplement: S4 Table — (DOCX) [file pone.0246095.s007.docx]

**S4 Table.** SDS sedimentation of NILs in comparison with parents (year 1).

| **SDSS (Year 1)** | | | | |
| --- | --- | --- | --- | --- |
| **C306/4*PBW343** | |  | **C306/4*PBW621** | |
| **NILC3A** | 3.5±0.1^d^ |  | **NILC6A** | 3.63±0.08^b^ |
| **NILC3B** | 2.43±0.17^a^ |  | **NILC6B** | 3.9±0^b^ |
| **NILC3C** | 2.5±0.03^ab^ |  | **NILC6C** | 6.1±0.18^de^ |
| **NILC3D** | 2.9±0.03^bc^ |  | **NILC6D** | 6.33±0.1^e^ |
| **NILC3E** | 5.13±0.11^e^ |  | **NILC6E** | 4.87±0.05^c^ |
| **NILC3F** | 2.6±0.06^abc^ |  | **NILC6F** | 5.47±0.3^cd^ |
| **NILC3G** | 2.93±0.02^bc^ |  | **NILC6G** | 7.07±0.04^f^ |
| **NILC3H** | 3±0^c^ |  | **NILC6H** | 5.5±0.12^cd^ |
| **C306** | 2.27±0.08^a^ |  | **C306** | 2.27±0.08^a^ |
| **PBW343** | 6.6±0.05^f^ |  | **PBW621** | 9.77±0.05^g^ |

Data was represented in mean ± SE of 3 replicates. Same letters depict they are not significantly different (p<0.05)
