# Supplementary material for: A null allele of granule bound starch synthase (Wx-B1) may be one of the major genes controlling chapatti softness
Source: PLoS One. 2021 Jan 28;16(1):e0246095. doi: 10.1371/journal.pone.0246095 (PMC7842929; doi:10.1371/journal.pone.0246095)
Supplement: S5 Table — (DOCX) [file pone.0246095.s008.docx]

**S5 Table.** Dough extensibility of NILs in comparison with parents (year 1).

| **Dough Extensibility Parameters (Year 1)** | | | |
| --- | --- | --- | --- |
| **ID** | **Separating Distance** | **Peak Positive Force (g)** | **Area to positive peak (g.s)** |
| **NILC3A** | 10.69±0.26^d^ | 36.55±0.29^g^ | 114.8±4.52^e^ |
| **NILC3B** | 7.91±0.09^b^ | 13.69±0.07^b^ | 56.39±1.2^a^ |
| **NILC3C** | 10.02±0.13^cd^ | 25.17±0.1^e^ | 100.04±3.68^de^ |
| **NILC3D** | 8.26±0.24^b^ | 15.18±0.07^c^ | 61.18±1.55^ab^ |
| **NILC3E** | 12.96±0.25^e^ | 61.02±0.13^h^ | 195.64±5.97^f^ |
| **NILC3F** | 10.09±0.02^cd^ | 24.67±0.45^e^ | 90.14±4.26^cd^ |
| **NILC3G** | 9.45±0.17^c^ | 30.84±0.51^f^ | 114.3±6.42^e^ |
| **NILC3H** | 5.63±0.16^a^ | 10.85±0.08^a^ | 44.38±0.51^a^ |
| **C306** | 9.51±0.01^c^ | 21.33±0.07^d^ | 79.45±0.02^bc^ |
| **PBW343** | 10.48±0.04^d^ | 31.92±0.31^f^ | 106.64±0.48^de^ |
| **NILC6A** | 7.36±0.22^a^ | 13.56±0.45^a^ | 62.31±3.09^a^ |
| **NILC6B** | 13.57±0.36^c^ | 32.55±0.5^e^ | 132.99±6.32^c^ |
| **NILC6C** | 13.7±0.41^c^ | 26.34±0.62^d^ | 154.31±3.04^d^ |
| **NILC6D** | 12.58±0.32^c^ | 19.91±0.18^c^ | 123.07±0.86^c^ |
| **NILC6E** | 12.36±0.03^c^ | 16.03±0.2^b^ | 94.85±4.18^b^ |
| **NILC6F** | 13.19±0.1^c^ | 28.09±0.21^d^ | 123.21±4.65^c^ |
| **NILC6G** | 12.29±0.32^c^ | 21.04±0.14^c^ | 120.32±3.84^c^ |
| **NILC6H** | 8.29±0.43^ab^ | 17.16±0.32^b^ | 74.33±0.84^a^ |
| **C306** | 9.51±0.01^b^ | 21.33±0.07^c^ | 79.45±0.02^ab^ |
| **PBW621** | 13.46±0.08^c^ | 39.16±0.29^f^ | 168.46±0.81^d^ |

Data was represented in mean ± SE of 6 replicates. Same letters depict they are not significantly different (p<0.05).
