# Supplementary material for: A null allele of granule bound starch synthase (Wx-B1) may be one of the major genes controlling chapatti softness
Source: PLoS One. 2021 Jan 28;16(1):e0246095. doi: 10.1371/journal.pone.0246095 (PMC7842929; doi:10.1371/journal.pone.0246095)
Supplement: S6 Table — (DOCX) [file pone.0246095.s009.docx]

**S6 Table.** Solvent retention capacity of NILs in comparison with parents (year 1).

| **Solvent Retention Capacity (Year 1)** | | | | | |
| --- | --- | --- | --- | --- | --- |
| **ID** | **Water SRC** | **Na_2_CO_3_ SRC** | **Lactic acid SRC** | **Sucrose SRC** | **GPI** |
| **NILC3A** | 160.4±0.43^g^ | 156.23±10.81^e^ | 175.92±0.98^e^ | 144.77±2.65^de^ | 0.59±0.03^c^ |
| **NILC3B** | 140.17±0.44^a^ | 136.5±0.29^abc^ | 160.17±0.83^abc^ | 132±2^b^ | 0.6±0^c^ |
| **NILC3C** | 150.67±0.19^de^ | 154.32±6.78^de^ | 164.33±0.21^c^ | 136.32±1.09^bc^ | 0.57±0.02^bc^ |
| **NILC3D** | 152.75±0.58^ef^ | 133.33±6.23^ab^ | 170.25±0.23^cd^ | 143.52±2.04^de^ | 0.62±0.02^c^ |
| **NILC3E** | 140.32±0.24^a^ | 159.22±1.34^de^ | 159.37±0.73^abc^ | 148.62±1.37^e^ | 0.52±0^ab^ |
| **NILC3F** | 147.5±0.5^bc^ | 127.38±3.8^a^ | 150.67±10.67^a^ | 119.17±2.68^a^ | 0.61±0.05^c^ |
| **NILC3G** | 146.5±1.32^b^ | 143.67±4.13^abcd^ | 163±0.76^bc^ | 142±0.58^d^ | 0.57±0.01^bc^ |
| **NILC3H** | 149.32±1.74^cd^ | 146.65±5.72^bcde^ | 165.23±0.72^cd^ | 141.65±0.89^cd^ | 0.57±0.01^bc^ |
| **C306** | 140.17±0.44^a^ | 151.67±1.2^cde^ | 152.67±1.01^ab^ | 167±2.31^f^ | 0.48±0^a^ |
| **PBW343** | 153.83±0.33^f^ | 163.17±1.59^e^ | 170.5±0.87^cd^ | 170±0.87^f^ | 0.51±0.01^a^ |
| **NILC6A** | 147.17±0.1^bcd^ | 156.83±0.95^bc^ | 160.67±0.35^b^ | 150.5±0.6^bc^ | 0.52±0^c^ |
| **NILC6B** | 144.5±0.6^bc^ | 151.5±1.45^ab^ | 168.33±0.54^cd^ | 152.33±0.59^bc^ | 0.55±0.01^d^ |
| **NILC6C** | 146.4±0.26^bcd^ | 168.07±0.94^d^ | 166.32±0.73^c^ | 150.42±0.92^bc^ | 0.52±0^c^ |
| **NILC6D** | 147.83±0.19^bcde^ | 151±0.5^ab^ | 167.67±0.25^c^ | 134.83±0.98^a^ | 0.59±0^e^ |
| **NILC6E** | 148.83±0.82^def^ | 149.83±1.58^a^ | 167.33±0.67^c^ | 134±0.33^a^ | 0.59±0.01^e^ |
| **NILC6F** | 148.33±1.38^cdef^ | 159.78±0.6^c^ | 165.92±0.12^c^ | 154.43±1.31^c^ | 0.53±0^c^ |
| **NILC6G** | 144.17±0.38^b^ | 149.17±0.69^a^ | 167.17±0.77^c^ | 147.17±0.59^b^ | 0.56±0^d^ |
| **NILC6H** | 152±1.44^f^ | 151.17±1.95^ab^ | 168.67±1.08^cd^ | 137.33±2.07^a^ | 0.58±0^e^ |
| **C306** | 140.17±0.25^a^ | 151.67±0.69^ab^ | 152.67±0.59^a^ | 167±1.33^d^ | 0.48±0^a^ |
| **PBW621** | 150.83±0.19^ef^ | 161.83±1.62^c^ | 171.17±0.25^d^ | 180±0.5^d^ | 0.5±0^b^ |

Data was represented in mean ± SE of 3 replicates. Same letters depict they are not significantly different (p<0.05).
