# Supplementary material for: A null allele of granule bound starch synthase (Wx-B1) may be one of the major genes controlling chapatti softness
Source: PLoS One. 2021 Jan 28;16(1):e0246095. doi: 10.1371/journal.pone.0246095 (PMC7842929; doi:10.1371/journal.pone.0246095)
Supplement: S7 Table — (DOCX) [file pone.0246095.s010.docx]

**S7 Table.** Chapatti sensory parameters of NILs in comparison with parents (year1).

| **Year-1** | | | | | | | | | | | | |
| --- | --- | --- | --- | --- | --- | --- | --- | --- | --- | --- | --- | --- |
| **Sample ID** | **Stickiness** | **Rollability** | **Puffing** | **Black spots** | **Colour** | **Taste** | **Aroma** | **Mouth feel** | **Tearing** | **Softness** | **Total** | **TM at 0’ (M Pa)** |
| **NILC3A** | 7.4±0.16^bc^ | 7.9±0.1^ab^ | 7.8±0.13^ab^ | 6.2±0.13^ab^ | 6.8±0.13^b^ | 7±0^ab^ | 6.2±0.13^a^ | 7.2±0.13^b^ | 6.8±0.13^abc^ | 7.8±0.13^d^ | 71.1±0.31^c^ | 0.41±0.02^c^ |
| **NILC3B** | 6.4±0.16^a^ | 8.2±0.13^abc^ | 7.2±0.25^ab^ | 7.4±0.34^cd^ | 6.2±0.13^ab^ | 7.2±0.33^ab^ | 6.2±0.13^a^ | 7.8±0.29^bc^ | 7.4±0.16^bc^ | 7.2±0.13^cd^ | 71.2±0.71^c^ | 0.17±0.02^a^ |
| **NILC3C** | 7.8±0.13^c^ | 8.5±0.17^bc^ | 8.2±0.13^bc^ | 7.4±0.16^cd^ | 7.4±0.16^c^ | 7.2±0.13^ab^ | 7.8±0.13^b^ | 8.2±0.25^bcd^ | 8.8±0.13^b^ | 8.4±0.16^e^ | 79.7±0.56^e^ | 0.39±0.02^c^ |
| **NILC3D** | 7.6±0.16^c^ | 8.3±0.26^abc^ | 8.2±0.25^bc^ | 6.8±0.13^bc^ | 6.2±0.13^ab^ | 6.4±0.27^a^ | 6.8±0.42^a^ | 7.2±0.13^b^ | 7.2±0.13^bc^ | 7.2±0.13^cd^ | 71.9±0.64^c^ | 0.17±0.03^a^ |
| **NILC3E** | 7.4±0.16^bc^ | 7.8±0.13^ab^ | 7.8±0.13^ab^ | 7.8±0.13^d^ | 8.2±0.13^d^ | 7.8±0.25^b^ | 6.6±0.16^a^ | 8.4±0.31^cd^ | 7.4±0.27^bc^ | 7.8±0.13^d^ | 77±0.73^d^ | 0.35±0^bc^ |
| **NILC3F** | 6.4±0.16^a^ | 7.7±0.21^ab^ | 8.2±0.33^bc^ | 5.8±0.13^a^ | 6.8±0.13^b^ | 7±0.42^ab^ | 6±0.21^a^ | 7.8±0.25^bc^ | 7.6±0.16^c^ | 6.2±0.13^b^ | 69.5±0.72^bc^ | 0.36±0.01^bc^ |
| **NILC3G** | 6.9±0.1a^b^ | 7.4±0.16^a^ | 7.5±0.17^ab^ | 6.6±0.16^b^ | 5.8±0.13^a^ | 6.8±0.2^ab^ | 7.6±0.16^b^ | 7.5±0.22^bc^ | 7.5±0.45^bc^ | 7±0.21^c^ | 70.6±1.02^bc^ | 0.4±0.04^c^ |
| **NILC3H** | 6.4±0.16^a^ | 7.9±0.38^ab^ | 7.6±0.16^ab^ | 5.8±0.13^a^ | 6.2±0.13^ab^ | 7.2±0.13^ab^ | 6.2±0.13^a^ | 7.8±0.36^bc^ | 6.6±0.16^ab^ | 6.4±0.16^b^ | 68.1±0.74^b^ | 0.16±0^a^ |
| **C306** | 9±0^d^ | 9±0^c^ | 9±0^c^ | 8.8±0.13^e^ | 8.8±0.13^e^ | 9±0^c^ | 8.4±0.16^c^ | 9±0^b^ | 9±0^d^ | 9±0^f^ | 89±0^f^ | 0.26±0^ab^ |
| **PBW343** | 6.8±0.13^a^ | 7.8±0.25^ab^ | 7.4±0.16^ab^ | 6.2±0.13^ab^ | 6.4±0.16^b^ | 6.8±0.13^ab^ | 6.2±0.13^a^ | 6±0.3^a^ | 6.2±0.25^a^ | 5.6±0.16^a^ | 65.4±0.69^a^ | 0.26±0.01^ab^ |
| **NILC6A** | 7.5±0.22^a^ | 7.6±0.16^a^ | 7.8±0.25^abc^ | 6.8±0.13^a^ | 6.2±0.13^a^ | 6.6±0.16^a^ | 7.7±0.33^bcd^ | 7.2±0.13^ab^ | 8.2±0.29^abc^ | 7.8±0.13^a^ | 73.4±1.05^a^ | 0.35±0.09^ab^ |
| **NILC6B** | 7.4±0.16^a^ | 7.8±0.13^ab^ | 8.6±0.16^bcd^ | 8.2±0.13^cd^ | 7.2±0.13^b^ | 6.6±0.16^a^ | 7.6±0.22^bcd^ | 7.2±0.14^ab^ | 8.1±0.23^abc^ | 8.1±0.31^ab^ | 73.2±0.53^a^ | 0.22±0.04^a^ |
| **NILC6C** | 7.4±0.16^a^ | 7.4±0.27^a^ | 7.6±0.16^ab^ | 6.4±0.16^a^ | 6.2±0.13^a^ | 6.9±0.28^a^ | 7.9±0.38^bcd^ | 7.5±0.34^ab^ | 7.9±0.18^ab^ | 8.1±0.31^ab^ | 73.3±1.56^a^ | 0.2±0.01^a^ |
| **NILC6D** | 8±0.42^ab^ | 8.1±0.38^ab^ | 7.4±0.56^a^ | 6.6±0.16^a^ | 6.8±0.13^ab^ | 7.3±0.42^a^ | 7.5±0.43^abc^ | 8±0.3^bc^ | 8.1±0.31^abc^ | 8.1±0.31^ab^ | 75.9±2.76^a^ | 0.24±0^a^ |
| **NILC6E** | 7.2±0.13^a^ | 7.2±0.13^a^ | 8.6±0.16^bcd^ | 8.2±0.13^cd^ | 8.2±0.13^c^ | 8.4±0.27^b^ | 8.6±0.16^cd^ | 8.6±0.16^cd^ | 8.6±0.16^bc^ | 7.8±0.13^a^ | 81.4±0.65^b^ | 0.35±0.03^ab^ |
| **NILC6F** | 7.9±0.38^a^ | 7.6±0.16^a^ | 7.6±0.16^ab^ | 7.4±0.16^b^ | 6.4±0.16^a^ | 6.6±0.27^a^ | 6.4±0.16^a^ | 7.4±0.22^ab^ | 8.2±0.25^abc^ | 8.3±0.26^ab^ | 73.8±1.04^a^ | 0.48±0.03^b^ |
| **NILC6G** | 7.8±0.36^a^ | 7.2±0.29^a^ | 8.4±0.16^abcd^ | 6.6±0.16^a^ | 6.2±0.13^a^ | 7.3±0.52^a^ | 7.3±0.26^ab^ | 8±0.33^bc^ | 7.8±0.2^ab^ | 7.8±0.25^a^ | 74.4±1.9^a^ | 0.3±0.01^ab^ |
| **NILC6H** | 8.2±0.29^ab^ | 8.6±0.16^bc^ | 8.8±0.13^cd^ | 8.8±0.13^b^ | 8.8±0.13^c^ | 8.8±0.13^b^ | 8.8±0.13^d^ | 9±0^d^ | 9±0^c^ | 9±0^b^ | 87.8±0.57^c^ | 0.39±0.04^ab^ |
| **C306** | 9±0^b^ | 9±0^c^ | 9±0^d^ | 8.8±0.13^b^ | 8.8±0.13^c^ | 9±0^b^ | 8.4±0.16^bcd^ | 9±0^d^ | 9±0^c^ | 9±0^b^ | 89±0^c^ | 0.26±0^a^ |
| **PBW621** | 7±0.21^a^ | 7.6±0.27^a^ | 8.6±0.16^bcd^ | 7.8±0.25^bc^ | 6.4±0.34^a^ | 6.4±0.16^a^ | 7.4±0.34^abc^ | 6.6±0.16^a^ | 7.6±0.16^a^ | 7.4±0.27^a^ | 72.8±0.57^a^ | 0.27±0.01^a^ |

TM- Tensile Modulus. Data was represented in mean ± SE of 10 replicates. Same letters depict they are not significantly different (p<0.05)
