# Supplementary material for: A null allele of granule bound starch synthase (Wx-B1) may be one of the major genes controlling chapatti softness
Source: PLoS One. 2021 Jan 28;16(1):e0246095. doi: 10.1371/journal.pone.0246095 (PMC7842929; doi:10.1371/journal.pone.0246095)

Fig 2B. Screening of Indian germplasm with codominant PCR markers to understand the variation of GBSS alleles


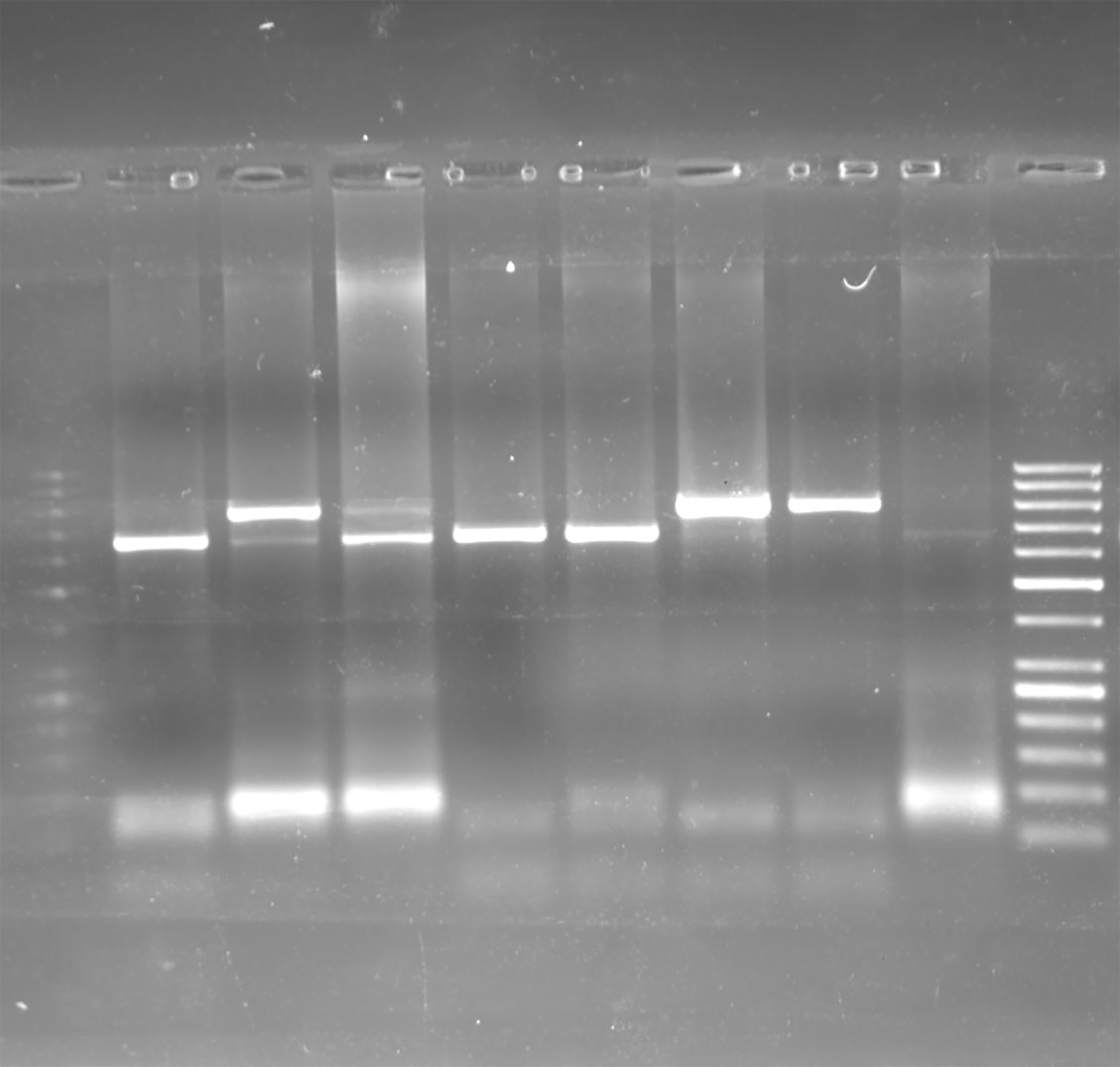


S1A Figure. **Gel-image of GBSS-7A (*Wx-A1*) between Indian cultivars**


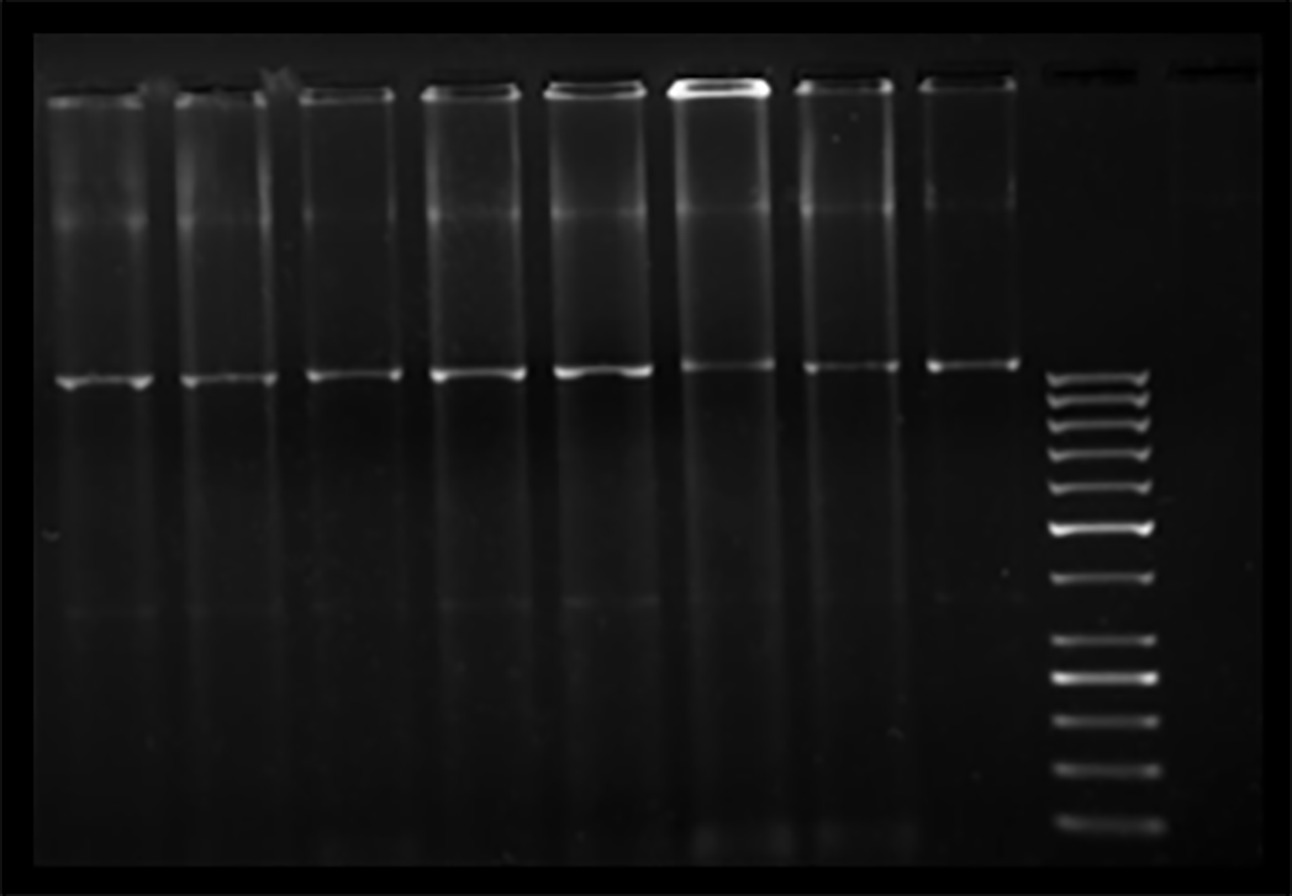


S1B Figure. **Gel-image of GBSS-7D (*Wx-D1*) between Indian cultivars**


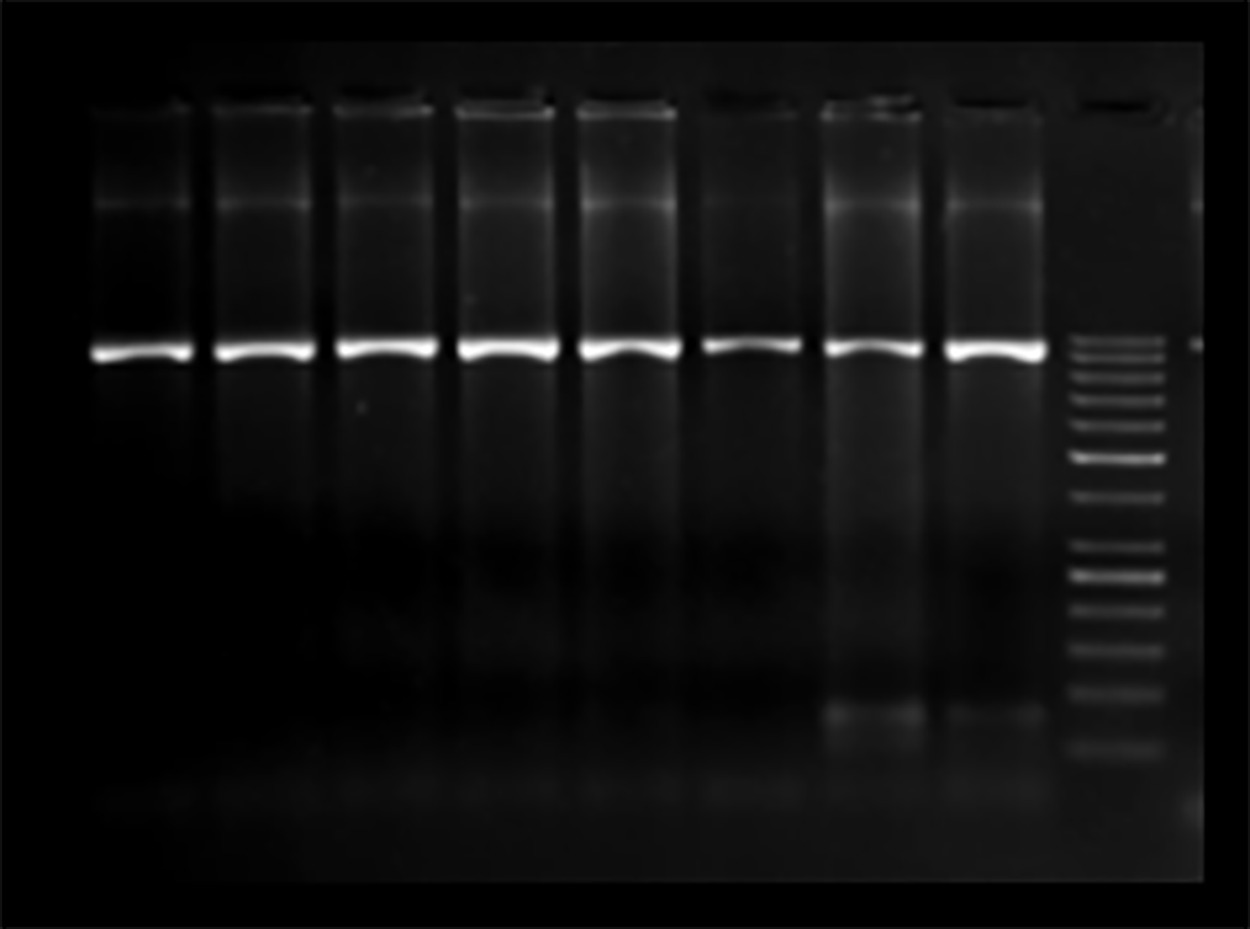

Supplement: S1 File — (DOCX) [file pone.0246095.s011.docx]
